# Effects of walking speed and slope on pedobarographic findings in young healthy adults

**DOI:** 10.1371/journal.pone.0220073

**Published:** 2019-07-24

**Authors:** Seungbum Koo, Moon Seok Park, Chin Youb Chung, Ji Soo Yoon, Chulhee Park, Kyoung Min Lee

**Affiliations:** 1 Department of Mechanical Engineering, Chung-Ang University, Seoul, Korea; 2 Department of Orthopaedic Surgery, Seoul National University Bundang Hospital, Kyungki, Korea; Virginia Tech, UNITED STATES

## Abstract

**Background:**

This study aimed to investigate the effects of walking speed and slope on foot pressure changes in young healthy adults.

**Methods:**

Twenty young healthy adults (mean age 22.4 years, SD 1.2 years; 10 male and 10 female) participated in the study. Dynamic pedobarographic data during treadmill walking were obtained for combinations of three different walking speeds (3.2 km/hr, 4.3 km/hr, and 5.4 km/hr) and 5 different slopes (downhill 8 degrees, downhill 4 degrees, ground walking (0 degree), uphill 4 degrees, and uphill 8 degrees). Pedobarographic data such as the peak pressure and pressure–time integral were measured on five plantar segments: medial forefoot (MFF), lateral forefoot (LFF), medial midfoot (MMF), lateral midfoot (LMF), and heel. Maximum ankle dorsiflexion was also recorded using the Plug in Gait marker set.

**Results:**

All participants maintained heel-toe gait in all walking conditions. The peak pressure on the MFF during downhill slope walking was lower than that during ground and uphill walking, whereas the peak pressure on the MFF during uphill slope walking was similar to that during ground walking at each walking speed. The peak pressures on the heel were similar for different walking slopes at each walking speed. The peak pressures on the MFF and heel increased with an increase in walking speed. The pressure-time integral of the MFF did not show significant changes at different walking speeds and slopes. The pressure-time integral of the heel increased with an increase in walking slope and decrease in walking speed.

**Conclusions:**

Different walking speeds and slopes affected the pedobarographic characteristics of young healthy adults. Downhill walking with slower speed appeared to be beneficial to reduce or optimize MFF pressures, while downhill walking at a comfortable speed would be helpful to reduce or optimize heel pressures. The findings of this study have clinical implications in recommending activities to patients with foot pressure-related symptoms and disorders.

## Introduction

Foot pressure has been reported to be associated with various foot symptoms and problems, including metatarsalgia, heel pain, callosity, diabetic foot ulcer, and foot deformities.[1–7] Increased plantar pressure and abnormal distribution of foot pressure could cause mechanical pain and biomechanical imbalance.[7,8] As pressure is defined as force per unit area perpendicular to the contact surface,[9] subject-related factors such as weight, area of contact, vertical acceleration of the foot at the time of ground contact, plantar soft tissue property, and shoe wear as well as other factors could possibly affect foot pressure.

Peak pressure and pressure-time integral are the most commonly used foot pressure measures used in clinical evaluation. Peak pressure is the maximum pressure, and it has been reported to be associated with foot pain, diabetic neuropathy, and the development of diabetic foot ulcers as well as foot discomfort in normal subjects.[10,11] The pressure-time integral is the accumulation of foot pressure according to contact time, and it is known to be associated with diabetic foot ulcers and foot pain in patients with cavus deformity.[12,13]

The effect of walking conditions on foot pressure has not been comprehensively understood. Walking speed and slope could significantly affect the foot pressure pattern and distribution. Although increased walking speed has been reported to increase foot pressure,[14,15] it has not been investigated in combination with walking slope. Knowledge on foot pressure changes according to various walking speeds and slopes would be useful in recommending activities for patients with pressure-related foot problems. Therefore, this study aimed to investigate the effects of various walking slopes and speeds on foot pressure changes in young healthy male and female adults.

## Methods

This prospective study was approved by the institutional review board of Chung-Ang University (IRB No. 1041078-201401-BM-002-02), and informed consent was obtained from all the participants.

### Subjects

Young adult volunteers without any medial or surgical problems limiting their daily activities were recruited. Of these volunteers, those with any of the following conditions were excluded from the study: 1) previous orthopedic surgery, 2) congenital anomaly, 3) neuromuscular disease, 4) orthopedic complaints such as pain or stiffness, 5) foot deformities, and 6) other condition that could limit their activities or comfortable walking. Ten male and 10 female young adults were finally included for participation in the study. Demographic data were collected, including age, sex, height, and weight. This study subjects was identical to those of our previsouly published article.[16]

### Maximum ankle dorsiflexion and foot pressure measurements

Kinematic data of the ankle were collected simultaneously with foot pressure measurement during treadmill walking. Four reflective skin markers were placed on the heel, dorsum of the second metatarsal head, lateral malleolus, and anterior shank by a single skilled operator according to the Plug in Gait marker set. Kinematic data of the ankle were captured at a sampling rate of 100 Hz by 6 motion capture cameras, and the data from three gait trials were averaged to a single value for each participant. The kinematic data were retrieved and digitally analyzed using a Vicon MX T-10 system (Vicon Motion Systems, Oxford, UK). The maximum ankle dorsiflexion was determined and recorded.

**Fig 1:**
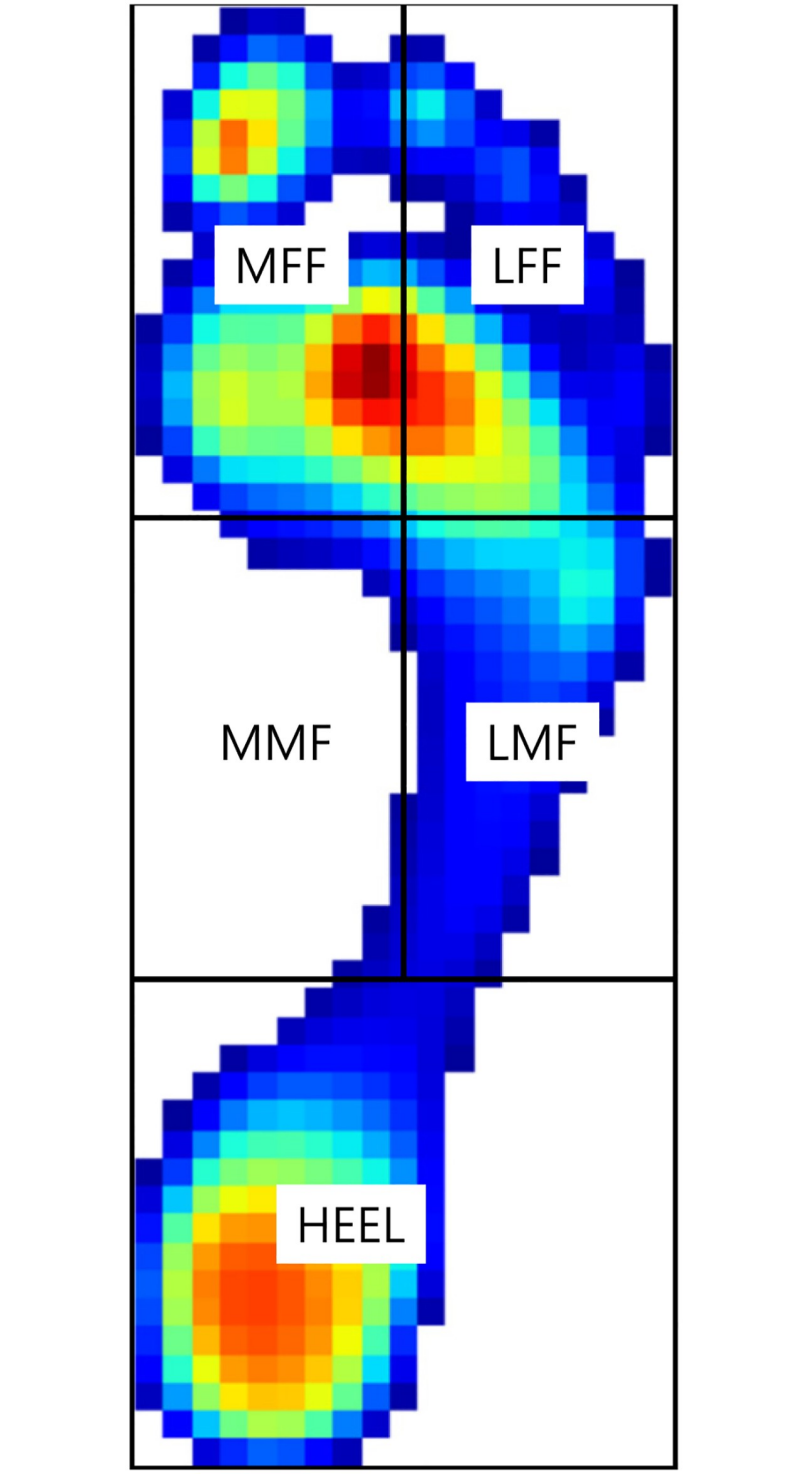
The dynamic pedobarograph was analyzed by dividing the plantar surface into five segments. The foot includes the anterior third, middle third, and posterior third. The anterior and middle thirds are further divided into medial and lateral segments, representing the medial forefoot (MFF), lateral forefoot (LFF), medial midfoot (MMF), and lateral midfoot (LMF). The posterior third represents heel segment.

Foot pressure was measured dynamically during barefoot walking using a treadmill pedobarographic system, FDM-TDSL (Zebris Medical, Isny Im Allgäu, Germany). The treadmill belt had a 150 × 50 cm^2^ running surface and 94.6 × 40.6 cm^2^ sensor surface, consisting of 5376 pressure-sensing cells/cm^2^. Data were obtained at a sampling rate of 100 Hz and were recorded using a computer and specific software. Each participant was instructed to walk barefoot on the treadmill pedobarographic system at 15 different combinations of walking speed and slope. The walking speed was set to 3.2 km/hr, 4.3 km/hr, or 5.4 km/hr. Slopes were downhill 8 degrees, downhill 4 degrees, ground walking (0 degrees), uphill 4 degrees, and uphill 8 degrees. The standard walking speed was considered to be 4.3 km/hr [17,18] and the standard slope, 0 degrees. The data of standard walking condition was used as a reference data for comparison, and was obtained from our previously published article.[16] Pedobarographic data were collected during 1-minute walking and averaged using specific software designed for the treadmill foot pressure measurement system. The area of pedobarographic measurement was divided into five segments, medial forefoot (MFF), lateral forefoot (LFF), medial midfoot (MMF), lateral midfoot (LMF), and heel, wherein the forefoot, midfoot, and heel were equally divided (Fig 1).[19,20] The peak pressure and pressure-time integral were obtained for each segment.

### Statistical analysis

Descriptive data summary was conducted including mean and standard deviation (SD). Data normality was tested using the Kolmogorov-Smirnov test. The effect of walking speed and slope on the pedobarographic data was tested using the generalized estimating equation (GEE), and pairwise comparisons were adjusted using Bonferroni correction. All statistical analyses were performed using SPSS software (version 20.0, IBM Corp., Armonk, NY, USA), and significance was set at p<0.05.

## Results

### Demographic data of the subjects

Data on 20 right feet from the 20 subjects (mean age 22.4 years, SD 1.2 years), 10 male (mean age 22.0 years, SD 1.4 years) and 10 female (mean age 22.8 years, SD 0.9 years) subjects, were included in the data analysis. The ten male subjects had a mean height of 172.4 cm (SD 3.7 cm), a mean weight of 70.9 kg (SD 5.4 kg), and a mean body mass index (BMI) of 23. 9 kg/cm^2^ (SD 1.8 kg/cm^2^). The ten female subjects had a mean height of 161.6 cm (SD 4.6 cm), a mean weight of 52.2 kg (SD 5.0 kg), and a mean BMI of 20.0 kg/cm^2^ (SD 1.9 kg/cm^2^).

### Kinematic data

All participants maintained heel-toe gait in all walking conditions. The mean maximum dorsiflexion of the ankle was 17.6° (SD 5.6°) at a walking speed of 4.3 km/hr and 0 degree slope. Slower walking speed and slope walking (uphill or downhill) tended to increase the maximum ankle dorsiflexion during gait. The maximum dorsiflexion significantly increased at 3.2 km/hr and 8 degrees (p<0.001), 4.3 km/hr and 8 degrees (p<0.001), and 5.4 km/hr and 8 degrees (p = 0.008) compared to that at 4.3 km/hr and 0 degrees (Table 1).

**Table 1:** Maximum ankle dorsiflexion according to different walking speeds and slopes.

		Walking speed (km/hr)			Marginal mean
		3.2	4.3	5.4	
Walking slope (°)	8	25.7 (SD 7.1)* (p<0.0001)	24.8 (SD 6.2)* (p<0.001)	22.4 (SD 8.1)* (p = 0.008)	24.7 (SD 6.5)
	4	21.8 (SD 8.0) (p = 0.052)	20.7 (SD 7.2) (p = 0.647)	18.8 (SD 6.4) (p = 1.000)	20.4 (SD 7.2)
	0	18.7 (SD 7.8) (p = 1.000)	17.6 (SD 5.9)	16.6 (SD 6.9) (p = 1.000)	17.6 (SD 6.8)
	-4	19.8 (SD 8.4) (p = 1.000)	19.8 (SD 8.4) (p = 1.000)	15.5 (SD 6.6) (p = 1.000)	18.4 (SD 8.0)
	-8	20.5 (SD 8.3) (p = 1.000)	19.2 (SD 7.8) (p = 1.000)	18.7 (SD 7.4) (p = 1.000)	19.5 (SD 7.7)
Marginal mean		21.3 (SD 8.1)	20.4 (SD 7.4)	18.6 (SD 7.1)	20.1 (SD 7.6)

SD, standard deviation

Unit, degrees

p-value, pairwise comparisons with that at 4.3 km/hr and 0 degrees

*, statistical signficance

### Summary of pedobarographic data

The mean peak pressure was 33.2 N/cm^2^ (SD 9.0 N/cm^2^) on the MFF, 28.6 N/cm^2^ (SD 4.5 N/cm^2^) on the heel, 21.4 N/cm^2^ (SD 6.8 N/cm^2^) on the LFF, 18.2 N/cm^2^ (SD 8.5 N/cm^2^) on the MMF, and 15.0 N/cm^2^ (SD 6.5 N/cm^2^) on the LMF at a walking speed of 4.3 km/hr and 0 degree slope. The mean pressure-time integral was 182479.0 (SD 40353.4) for the heel, 169048.0 (SD 62285.4) for the MFF, 111648.0 (SD 58635.9) for the LFF, 94454.0 (SD 35291.3) for the LMF, and 27671.9 (SD 13176.5) for the MMF at a walking speed of 4.3 km/hr and 0 degree slope.

### Effect of walking speed and slope on the peak pressure of MFF

The peak pressure on MFF was significantly affected by walking speed (estimate 15.016, p = 0.001) and slope (estimate 76.389, p<0.001) as well as by the combination of walking speed and slope (estimate 16.419, p = 0.037). Faster walking slope and greater walking slope tended to increase the peak pressure on MFF (Table 2).

**Table 2:** Peak pressures on MFF according to different walking speeds and slopes.

		Walking speed (km/hr)			Marginal mean
		3.2	4.3	5.4	
Walking slope (°)	8	28.8 (SD 6.4)* (p = 0.027)	33.8 (SD 9.1) (p = 1.000)	34.4 (SD 9.5) (p = 1.000)	32.3 (SD 8.7)
	4	30.0 (SD 7.1) (p = 0.622)	33.5 (SD 8.8) (p = 1.000)	36.5 (SD 10.4) (p = 0.401)	33.3 (SD 9.1)
	0	30.1 (SD 7.2) (p = 1.000)	33.2 (SD 9.0)	36.6 (SD 11.6) (p = 0.166)	33.3 (SD 9.7)
	-4	24.7 (SD 5.3)* (p = 0.001)	25.9 (SD 6.8)* (p<0.001)	28.4 (SD 7.6) (p = 0.071)	26.3 (SD 6.7)
	-8	23.0 (SD 4.7)* (p<0.001)	24.6 (SD 7.1)* (p<0.001)	27.8 (SD 4.9) (p = 0.186)	25.2 (SD 5.9)
Marginal mean		27.3 (SD 6.8)	30.2 (SD 9.0)	32.7 (SD 9.7)	30.1 (SD 8.8)

MFF, medial forefoot; SD, standard deviation

Unit, N/cm^2^

p-value, pairwise comparisons with that at 4.3 km/hr and 0 degrees

*, statistical signficance

### Effect of walking speed and slope on the peak pressure of heel

The peak pressure on the heel was significantly affected by both walking speed (estimate 51.435, p<0.001) and slope (estimate 29.020, p<0.001) but not by the combination of walking speed and slope (estimate 13.641, p = 0.092). Faster walking speed tended to increase the peak pressure on heel, but walking slope did not appear to change the peak pressure on the heel at each walking speed (Table 3).

**Table 3:** Peak pressures on heel according to different walking speeds and slopes.

		Walking speed (km/hr)			Marginal mean
		3.2	4.3	5.4	
Walking slope (°)	8	25.8 (SD 4.1) (p = 0.147)	28.1 (SD 4.7) (p = 1.000)	32.1 (SD 5.8) (p = 0.420)	28.6 (SD 5.5) (p = 1.000)
	4	25.7 (SD 3.6)* (p = 0.048)	27.4 (SD 4.7) (p = 1.000)	30.1 (SD 5.4) (p = 1.000)	27.7 (SD 4.9) (p = 0.003)
	0	26.8 (SD 4.0) (p = 1.000)	28.6 (SD 4.5)	32.7 (SD 5.5)* (p = 0.003)	29.4 (SD 5.3)
	-4	25.9 (SD 4.3) (p = 0.822)	28.8 (SD 4.3) (p = 1.000)	31.9 (SD 6.3) (p = 0.640)	28.9 (SD 5.6) (p = 1.000)
	-8	25.4 (SD 5.4) (p = 1.000)	28.6 (SD 7.4) (p = 1.000)	33.8 (SD 10.3) (p = 1.000)	29.3 (SD 8.5) (p = 1.000)
Marginal mean		25.9 (SD 4.3) (p<0.001)	28.3 (SD 5.1)	32.1 (SD 6.9) (p<0.001)	28.8 (SD 6.1)

*, statistical signficance

### Effect of walking speed and slope on the pressure-time integral of MFF

The pressure-time integral of the MFF was significantly affected by walking speed (estimate 6.250, p = 0.044) and slope (estimate 46.051, p<0.001) but not by the combination of walking speed and slope (estimate 9.371, p = 0.312). There was no prominent change in the pressure-time integral of the MFF across all walking speeds or slopes (Table 4).

**Table 4:** Pressure-time integral on MFF according to different walking speeds and slopes.

		Walking speed (km/hr)			Marginal mean
		3.2	4.3	5.4	
Walking slope (°)	8	15.6 (SD 5.7) (p = 1.000)	15.9 (SD 5.8) (p = 1.000)	15.5 (SD 5.5) (p = 1.000)	15.6 (SD 5.6) (p = 0.412)
	4	14.6 (SD 6.2) (p = 0.278)	15.6 (SD 6.2) (p = 1.000)	16.4 (SD 5.4) (p = 1.000)	15.4 (SD 5.9) (p = 0.004)
	0	17.2 (SD 8.4) (p = 1.000)	16.9 (SD 6.2)	17.5 (SD 6.1) (p = 1.000)	17.2 (SD 6.9)
	-4	14.7 (SD 6.7) (p = 0.686)	15.8 (SD 6.4) (p = 1.000)	16.4 (SD 6.4) (p = 1.000)	15.6 (SD 6.4) (p = 0.257)
	-8	16.3 (SD 5.7) (p = 1.000)	18.0 (SD 6.7) (p = 1.000)	17.1 (SD 6.5) (p = 1.000)	17.1 (SD 6.2) (p = 1.000)
Marginal mean		15.7 (SD 6.5) (p = 0.038)	16.4 (SD 6.2)	16.5 (SD 5.9) (p = 1.000)	16.1 (SD 6.3)

*, statistical signficance

### Effect of walking speed and slope on the pressure-time integral of heel

The pressure-time integral of the heel was significantly affected by walking speed (estimate 130.432, p<0.001) and slope (estimate 334.166, p<0.001) and by the combination of walking speed and slope (estimate 41.643, p<0.001). Slower walking speed and greater slope tended to increase the pressure-time integral of the heel (Table 5).

**Table 5:** Pressure-time integral on heel according to different walking speeds and slopes.

		Walking speed (km/hr)			Marginal mean
		3.2	4.3	5.4	
Walking slope (°)	8	27.1 (SD 6.0)* (p<0.001)	21.6 (SD 4.0)* (p<0.001)	19.1 (SD 3.1) (p = 1.000)	22.7 (SD 5.6)
	4	27.7 (SD 6.7)* (p<0.001)	20.9 (SD 4.7)* (p<0.001)	18.3 (SD 3.1) (p = 1.000)	22.3 (SD 6.4)
	0	22.0 (SD 5.1)* (p<0.001)	18.2 (SD 4.0)	16.2 (SD 3.1)* (p<0.001)	18.8 (SD 4.8)
	-4	19.2 (SD 5.2) (p = 1.000)	15.8 (SD 4.0) (p = 0.436)	14.0 (SD 3.1)* (p<0.001)	16.3 (SD 4.7)
	-8	14.7 (SD 5.1)* (p = 0.015)	11.9 (SD 4.2)* (p<0.001)	11.1 (SD 4.0)* (p<0.001)	12.6 (SD 4.7)
Marginal mean		22.2 (SD 7.4)	17.7 (SD 5.4)	15.7 (SD 4.4)	18.5 (SD 6.5)

SD, standard deviation

Unit, N·s/cm^2^

p-value, pairwise comparisons with that at 4.3 km/hr and 0 degrees

*, statistical signficance

## Discussion

The peak pressure and pressure-time integral of the MFF and heel were significantly affected by both walking slope and speed. However, the effect of the combination of walking slope and speed on dynamic plantar pressure appeared to be more complex and unpredictable, evidenced by the inconsistent statistical significance on pairwise comparison with standard walking (4.3 km/hr and 0 degrees).

### Effect of walking speed and slope on the peak pressure of MFF

The peak pressures on the MFF and heel tended to increase with an increase in walking speed for each slope. The peak pressures on the MFF during downhill slope walking was smaller than that during ground walking, whereas the peak pressures on the MFF during uphill slope walking appeared to be similar to that of ground walking at each walking speed. On pairwise comparison, combinations of downhill slope and slower walking speed (at 3.2 km/hr and downhill 4 degrees, 4.3 km/hr and downhill 4 degrees, 3.2 km/hr and downhill 8 degrees, and 4.3 km/hr and downhill 8 degrees) showed significant decreases in the peak pressure on the MFF compared to that on standard walking (4.3 km/hr and 0 degrees). The combination of the greatest uphill slope (8 degrees) and slowest speed (3.2 km/hr) also caused a significant decrease in peak pressure on the MFF. Therefore, to reduce the peak pressure on the MFF, slower walking speed and downhill walking while maintaining heel-toe gait would be recommended.

The findings on the effect of walking speed on the peak pressure of the MFF are consistent with those of previous studies, which reported increased peak pressure on the MFF according to increased speed during jogging and walking.[14,15,21,22] Uphill slope walking did not increase the peak pressure on the MFF according to this study, and jogging with increasing slope significantly decreased the peak pressure on the MFF according to a previous study.[22] We consider that increased ankle dorsiflexion as a compensatory movement maintained or decreased the forefoot pressure although uphill walking possibly increased the forefoot pressure. Further study is required to determine the limit of uphill slope at which ankle dorsiflexion would not compensate for increased forefoot load.

### Effect of walking speed and slope on the peak pressure of heel

A faster walking speed increased the peak pressure on the heel, but the effect of walking slope on the peak pressure on the heel was not prominent at each walking speed. The peak pressure on the heel would be achieved at the initial contact of heel, which is heel strike of the stance phase. It is considered that heel contact according to different walking slopes did not affect the peak pressure, whereas the walking speed affected the force exerted on heel contact during gait.

### Effect of walking speed and slope on the pressure-time integral of MFF

The pressure-time integral on MFF did not show significant changes throughout all tested walking slopes and speeds in this study. It is considered that the change of walking slopes and speeds were appropriately compensated by complex effects of contact time and average pressure on MFF. The adaptive ankle dorsiflexion and muscle action would play an important role and this issue need to be investigated in a future study.

### Effect of walking speed and slope on the pressure-time integral of heel

The pressure-time integral of the heel tended to decrease with faster walking speed and downhill slope. On pairwise comparison, the majority of walking conditions (combinations of different walking slopes and speeds) showed significant differences from standard walking (4.3 km/hr and 0 degrees). Considering that the peak pressures on the heel tended to increase with faster walking speed, the effect of walking speed on heel pressure is controversial.

### Clinical implications

In clinical consideration of this study results, downhill walking with slower walking speed is recommended for those having forefoot problems associated with increased foot pressure. This is because downhill walking and slower walking speed appears to be beneficial in minimizing peak pressures on MFF, and pressure-time integral was not affected by any of walking slopes and speeds in our experimental condition. For those having heel problems caused by increased pressure, downhill walking with comfortable walking speed (4.3 km/hr) could be recommended. Peak pressure on heel decreased with slower walking speed but was not affected by walking slope. Pressure-time integral on heel decreased by downhill walking and faster walking speed. Therefore, compromising optimal peak pressure and pressure-time integral on heel, downhill walking with mid-walking speed is favorable. As of now, evidence is lacking which specific conditions are more closely related with either peak pressure or pressure-time integral. This issue needs more investigation for physicians to give more detailed recommendation to their patients.

Therefore, from the change in peak pressure and the pressure-time integral according to different walking slopes and speeds, it is acknowledged that increasing walking speeds increase peak pressures on the MFF and heel and slower walking speeds might have increased the contact time of the MFF and heel. In addition, downhill walking might increase the contact time of the MFF without increasing its peak pressure. We believe that the peak pressure on the MFF was compensated by increased ankle dorsiflexion until 8 degrees of uphill slope.

### Study limitations

This study has a few limitations. First, the range of walking speeds and slopes were slightly narrow and might not be representative of all clinical conditions. Therefore, the findings on the changes in dynamic plantar pressure according to walking slope and speed are confined to this study setting and are not generalizable. Second, treadmill walking could be different from ground walking in terms of biomechanics. Therefore, the study results might not be applicable to the ground walking condition. Third, the sample size was small, and generalization of the study results needs more extensive investigation.

## Conclusions

In summary, each of walking slope and speed significantly affected the foot pressure of the forefoot and heel, but the effect of the combination appeared to be complex and inconsistent. The peak pressure of the forefoot and heel did not increase (decrease or remain similar) prominently during uphill and downhill walking, although higher speed increased the peak pressures of both the forefoot and heel. Thus, the peak pressures of the forefoot and heel appeared to be compensated by increased ankle dorsiflexion during slope walking as long as the participants maintained heel-toe gait. Further investigation on the pressure pattern and contact time as well as pressure-related symptoms is required and future investigations should focus on kinematic study and compensation of foot pressure.

## Supporting information

S1 Table Raw data of the 20 subjects including demographic and pedobarographic data. (XLSX)
