# Correction: Effects of walking speed and slope on pedobarographic findings in young healthy adults

**DOI:** 10.1371/journal.pone.0221945

**Published:** 2019-08-27

**Authors:** Seungbum Koo, Moon Seok Park, Chin Youb Chung, Ji Soo Yoon, Chulhee Park, Kyoung Min Lee

There is an error in affiliation 1 for author Seungbum Koo. The correct affiliation 1 is: Department of Mechanical Engineering, Korea Advanced Institute of Science and Technology (KAIST), Daejon, Korea.
